# Organizational Support and Work Engagement in General Intensive Care Unit Nurses: The Double‐Edged Sword Effect of Emotional Labor

**DOI:** 10.1155/jonm/4743882

**Published:** 2026-03-04

**Authors:** He Li, Jing Wang, Heng Dai, Hailong Hou, Xianjuan Cheng, Tongtong Fu, Shiqi Xiao

**Affiliations:** ^1^ Department of Nursing, Shengjing Hospital of China Medical University, Shenyang, China, cmu.edu.cn; ^2^ Department of Nursing (Department of Intensive Care Unit), Shengjing Hospital of China Medical University, Shenyang, China, cmu.edu.cn; ^3^ Department of Nursing (Department of Nephrology), Shengjing Hospital of China Medical University, Shenyang, China, cmu.edu.cn; ^4^ Inpatient Department, Shengjing Hospital of China Medical University, Shenyang, China, cmu.edu.cn

**Keywords:** emotional labor, ICU nurses, organizational support, work engagement

## Abstract

**Objectives:**

Organizational support significantly influences nurses’ work engagement, but how it affects the engagement of general intensive care unit (GICU) nurses through distinct emotional labor strategies remains unclear. This study aims to find how emotional labor influences the effect of organizational support on work engagement in GICU nurses.

**Methods:**

This cross‐sectional survey was conducted from June to July 2024, involving 215 GICU nurses from five tertiary hospitals in Northeast China. Data were collected using demographic variable forms, emotional labor scales, nurse organizational support scales, and work engagement scales, and a structural equation model was established to analyze the relationships among emotional labor, organizational support, and work engagement in GICU nurses.

**Results:**

Surface acting was negatively correlated with organizational support and work engagement, while deep acting, natural acting, and organizational support were positively correlated with work engagement (*p* < 0.05). The mediating effects of organizational support on work engagement were statistically significant through surface acting (*β* = 0.099, 95% CI [0.004, 0.219]) and through the combined pathways of deep and natural acting (*β* = 0.211, 95% CI [0.054, 0.539]).

**Conclusions:**

Emotional labor exhibits a double‐edged sword effect in GICU nurses. Nursing managers can enhance nurses’ work engagement by implementing relevant organizational support measures to reduce surface acting while promoting deep acting and natural acting.

## 1. Introduction

The ICU, a department responsible for critically ill patients without accompanying caregivers, is a core component of the public health emergency system; its operational efficiency directly impacts the success rate of emergency medical interventions [1, 2]. The general intensive care unit (GICU) is representative of all ICU types, serving as a central location for treating critically ill patients from various departments and reflecting the concept of multidisciplinary collaborative treatment [3]. GICU nurses, as the backbone of daily operations, are responsible for implementing medical plans, monitoring patient conditions, and executing emergency interventions. Their work embodies the core characteristics of critical care medicine and meets the high standards of modern medical systems for critical patient care [4].

The daily work of GICU nurses is characterized by high complexity, high uncertainty, and high emotional intensity [5]. They must rapidly execute medical interventions in emergency situations and provide emotional support to patients and their families. This sustained high‐pressure environment consumes significant physical and mental resources [6, 7]. In such a high‐pressure and complex work environment, the ability of nurses to maintain high work engagement is crucial for personal efficacy and patient outcomes [8, 9]. Studies show that nurses with high work engagement are more likely to proactively optimize nursing processes, reduce medical errors, express a stronger willingness to remain in their positions, thrive at work, and improve job performance. These outcomes are vital for both patient safety and nursing team development [10–13].

As research on work engagement in GICU nurses deepens, increasing attention has been paid to external environmental factors, particularly organizational support, in shaping nurses’ work engagement. Studies indicate that organizational support can reduce negative emotions in nurses, enhance emotional intelligence and psychological resilience, and improve positive emotions and empathy fatigue and thereby increase work engagement [14, 15]. Nurses with high perceived organizational support demonstrate stronger psychological resilience in stressful job situations. This enhanced resilience fosters deeper job commitment, creating a supportive organizational environment. Such positive interactions lead to psychological safety driven by organizational identification, strengthening nurses’ sense of agency and willingness to innovate in clinical decision‐making, ultimately enhancing work engagement [16, 17].

GICU nurses frequently handle complex interpersonal interactions that require them to display emotions consistent with the expectations of society, hospitals, patients, families, and colleagues. This is the essence of emotional labor in nursing practice. Emotional labor is a significant component of clinical work for nurses [18]. Appropriate emotional labor strategies can improve communication skills and foster good nurse–patient relationships and departmental atmospheres and thereby enhance clinical nursing work [19]. Emotional labor is structured into three dimensions: surface acting, deep acting, and natural acting. These refer to (1) forced performance of emotions required by the organization, (2) cognitive restructuring to achieve expected emotional expression, and (3) maintaining consistency between internal feelings and external expressions. Natural acting differs from surface and deep acting in the authenticity of the expressed emotions [20]. Deep acting differs from surface acting in that it involves voluntary expression of the required emotions [21]. Studies show that healthcare professionals’ perceived organizational support is negatively related to surface acting and positively related to deep acting and natural acting [22]. Surface acting can damage doctor–patient relationships, reduce patient satisfaction, and lead to emotional exhaustion, depersonalization, stress, and burnout in nurses, affecting professional values and honor and thereby reducing job satisfaction and work engagement. Conversely, deep acting and natural acting promote positive emotional activation, maintain a high sense of achievement and self‐worth by timely mobilizing psychological resources, and enhance work engagement [15, 23].

When analyzing the pathways through which organizational support affects work engagement, the job demands‐resources (JD‐R) model is often used as a theoretical foundation. First proposed by Bakker and Demerouti in 2001, this model emphasizes the impact of resources and job demands on work engagement, with emotional demands being a key part of job demands [24–26]. Emotional labor can meet emotional job demands and provide cognitive and motivational resources, making it a part of personal resources [27]. Studies show that the most important component of job resources is organizational support [28]. Research further indicates that job resources can indirectly enhance work engagement through individual resources such as psychological capital and emotional regulation efficacy, with these individual resources showing significant mediating effects in the mechanism linking job resources to engagement [29]. Within this framework, emotional labor can meet emotional job demands and provide cognitive and motivational resources, thereby functioning as a personal resource that contributes to nurses’ engagement.

A double‐edged sword implies that one edge faces the enemy while the other faces oneself; it can help us overcome difficulties, but it can also cause us trouble. From the perspective of the JD‐R model, emotional labor can play both a depleting and an enriching role. Surface acting represents a high emotional demand that requires individuals to suppress or fake emotions inconsistent with their true feelings, thereby consuming psychological resources and inducing emotional exhaustion. In contrast, deep acting and natural acting function as personal resources that enable nurses to align emotional expression with inner experience, activate positive emotions, and maintain psychological resilience [30]. This mechanism forms the theoretical basis of the “double‐edged sword effect” of emotional labor, where different emotional labor strategies exert opposite influences on work engagement depending on whether they drain or replenish emotional resources [31].

Although existing studies have confirmed the importance of organizational support and emotional labor in shaping nurses’ work engagement, most of this evidence has been derived from general hospital wards such as oncology, pediatrics, or internal medicine [23, 32]. To date, empirical validation of these mechanisms in intensive care settings remains scarce, particularly within GICU, where the absence of family caregivers, the need for multidisciplinary collaboration, and the high frequency of emergencies create a uniquely demanding environment. These contextual differences suggest that the mechanisms linking organizational support, emotional labor, and work engagement observed in other specialties may not be directly applicable to GICU [33, 34].

Furthermore, while previous research has often examined emotional labor as a unidimensional construct, there is a lack of clarity on whether the three distinct strategies—surface acting, deep acting, and natural acting—play parallel mediating roles in this relationship [35–38]. Specifically, whether the double‐edged sword effect of emotional labor—where surface acting is detrimental while deep and natural acting are beneficial—holds true among GICU nurses remains underexplored [39–41]. Therefore, this study addresses these gaps by providing multicenter empirical evidence from 215 GICU nurses across five tertiary hospitals in Northeast China, examining the parallel mediating effects of surface acting, deep acting, and natural acting in the relationship between organizational support and work engagement, and grounding the analysis in the JD‐R model to link organizational‐level resources with individual emotional strategies that explain work engagement in a high‐intensity critical care context.

Thus, based on the identified theoretical framework and research gaps, the following hypotheses are proposed:

 H1: Organizational support negatively predicts surface acting in GICU nurses.
 H2: Organizational support positively predicts deep acting and natural acting in GICU nurses.
 H3: Organizational support positively predicts work engagement in GICU nurses.
 H4: Surface acting negatively predicts work engagement in GICU nurses.
 H5: Deep acting and natural acting positively predict work engagement in GICU nurses.
 H6: Surface acting, deep acting, and natural acting mediate the relationship between organizational support and work engagement in GICU nurses.

**FIGURE 1:**
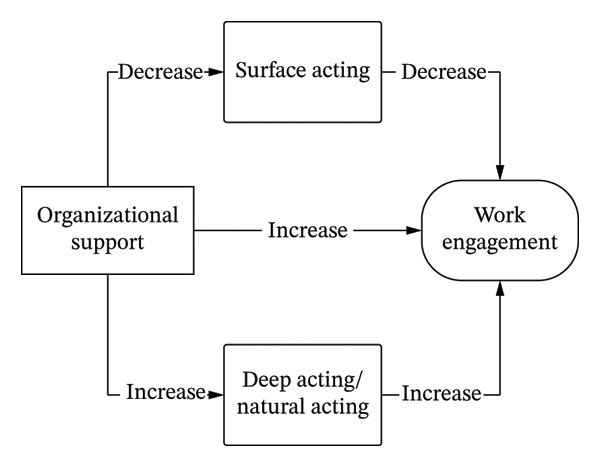
Hypothesized model of organizational support, emotional labor strategies, and work engagement in GICU nurses.

Based on the six hypotheses mentioned above, we incorporated them into a theoretical framework and established a hypothesized model. This model illustrates the direct relationship between organizational support and work engagement, as well as the parallel mediating effects of surface acting, deep acting, and natural acting. The hypothesized model is presented in Figure 1, which visually depicts how emotional labor strategies may influence the effect of organizational support on work engagement in GICU nurses.

## 2. Methods

### 2.1. Design

This cross‐sectional study adheres to the Strengthening the Reporting of Observational Studies in Epidemiology (STROBE) guidelines.

### 2.2. Study Objects

From June to July 2024, a convenience sampling method was used to select GICU nurses from five tertiary hospitals in Northeast China for questionnaire surveys. All these hospitals are large‐scale tertiary Grade A institutions, located in Shenyang, Jinzhou, Dalian, and Harbin, respectively.

Inclusion criteria were defined as follows: (1) possession of a nurse practice certificate and (2) at least 6 months of work experience in a GICU.

Exclusion criteria were defined as follows: (1) absence from clinical work for 2 weeks or more and (2) nurses who are interns, trainees, or rotational staff.

### 2.3. Sample Size

Given the use of structural equation modeling in this study, a sample size of no less than 200 cases is required [42]. Considering invalid samples and sample loss rates, the sample size should be increased by 20%, resulting in a total sample size of 250 for this study.

### 2.4. Research Tools

Self‐designed general data collection form: a general data collection form was designed based on research needs, including gender, age, ethnicity, education level, technical title, marital status, employment type, ICU work experience, monthly night shifts, and monthly income.

Emotional labor scale: this study adopted the emotional labor scale developed by Diefendorff based on Grandey’s scale [43], translated by Qiaoyang Bo. It includes three dimensions: surface acting, deep acting, and natural acting, with a total of 13 items. Scores range from 1 “*strongly disagree*” to 5 “*strongly agree*”. Higher scores indicate a higher level of emotional labor. Cronbach’s alpha coefficients for the three dimensions were 0.750, 0.721, and 0.718, respectively.

Nurse organizational support scale: this scale, derived from Eisenberger’s work [44], was translated and revised by Zhixia Chen and nursing specialized by Zuo Hongmei. It includes two dimensions: emotional support and instrumental support, with a total of 13 items. Scores range from 1 “*strongly disagree*” to 5 “*strongly agree*”. Higher scores indicate a higher level of organizational support. Cronbach’s alpha coefficient for the scale was 0.90, with 0.89 for the emotional support dimension and 0.68 for the instrumental support dimension.

Work engagement scale: the work engagement scale was developed by Schaufeli et al. in 2002 and translated and revised into Chinese by Yiwen Zhang et al. in 2005 [45]. It includes three dimensions: vigor, dedication, and absorption, with a total of 15 items. Scores range from 0 “never” to 6 “always”, with a total score of 0–90. Higher scores indicate a higher level of organizational support. Cronbach’s alpha coefficient for the scale was 0.947.

### 2.5. Data Collection

An electronic questionnaire was created using the electronic questionnaire platform. The questionnaire link and a brief study description were posted by the head nurses in WeChat groups; participation was voluntary. Questionnaires were administered during time periods when the subjects were relatively free. The questionnaire primarily used options instead of open‐ended questions, with mandatory fields and restrictions on the range of responses. Data were double‐entered. Questionnaires with short completion times, responses inconsistent with reality, or obvious and patterned answers were excluded. SPSS 27.0 was used to analyze each item, identify missing or invalid values, and cross‐verify with original questionnaires for correction. Additionally, 10% of the original questionnaires were randomly selected for verification to ensure data accuracy.

### 2.6. Ethical Approval

In accordance with research ethics and the principles of the Declaration of Helsinki, this study was approved by the Medical Ethics Committee of Shengjing Hospital of China Medical University on May 21, 2024, with the ethical number 2024PS1091K. Before including subjects in the study, a standardized informed consent process was conducted, including the study’s objectives, significance, and risks, and subjects signed consent forms to participate voluntarily.

### 2.7. Data Analysis

SPSS 27.0 and AMOS 28.0 statistical software were used to analyze the data.

First, descriptive statistics, *t*‐tests, and ANOVA were used to examine sample characteristics and group differences. Second, Pearson correlations were conducted to explore relationships among variables. Third, multiple regression analysis was performed as a preliminary test of the hypotheses before building the complex SEM model. Finally, SEM was employed as the primary method to test the integrated theoretical model and mediation effects simultaneously, providing a more robust test of our hypotheses. Descriptive statistics for demographic variables were presented as counts and percentages for categorical data and means and standard deviations for continuous data. The reliability of the questionnaires was evaluated using Cronbach’s α coefficient, and the validity was assessed using KMO values, Bartlett’s test of sphericity, and variance explained.

## 3. Results

### 3.1. General Data of GICU Nurses and Its Impact on Work Engagement

A total of 253 questionnaires were collected, and after excluding those with short answering times and unreasonable responses, 215 valid questionnaires were obtained, with an effective response rate of 84.98%; in these, 177 were females (82.3%); the average age was (33.54 ± 6.20) years, with the majority aged 30–39 (*n* = 124, 57.7%); the majority were Han (*n* = 187, 87%); most had a bachelor’s degree (*n* = 189, 87.9%); the majority were senior nurses (*n* = 88, 40.9%); most were married (*n* = 141, 65.6%); the majority were contract employees (*n* = 176, 81.9%); the average ICU work experience was (9.98 ± 6.68) years, with the majority having over 14 years of experience (*n* = 59, 27.4%); the average number of night shifts per month was (5.97 ± 2.56), with most having 6‐7 night shifts (*n* = 109, 50.7%); the average monthly income was (12831.63 ± 4540.59) yuan, with the majority earning 10001–15000 yuan (*n* = 68, 31.6%). All general data passed the K‐S normality test. The impact of general data on work engagement was tested using independent sample *t*‐tests and one‐way ANOVA, and the results showed that work engagement scores in GICU nurses differed significantly based on ICU work experience (*p* < 0.05). See Table 1 for details.

**TABLE 1:** General data and its impact on work engagement.

Item	Category	*n*	%	Work engagement ($\overset{¯}{x}$ ± SD)	*t/F* value	*p* value
Gender					1.789	0.075
	Male	38	17.7	4.04 ± 1.45		
	Female	177	82.3	3.65 ± 1.18		

Age					0.666	0.574
	21–29	57	26.5	3.84 ± 1.33		
	30–39	124	57.7	3.65 ± 1.22		
	40–49	32	14.9	3.81 ± 1.19		
	50–59	2	0.9	2.87 ± 0.37		

Ethnicity					0.77	0.464
	Han	187	87	3.73 ± 1.23		
	Manchu	22	10.2	3.75 ± 1.33		
	Others	6	2.7	3.10 ± 1.31		

Education level					2.035	0.110
	High school/secondary vocational	2	0.9	2.97 ± 0.52		
	Associate degree	18	8.4	4.36 ± 1.42		
	Bachelor’s	189	87.9	3.67 ± 1.22		
	Master’s and above	6	2.8	3.57 ± 0.84		

Technical title					1.231	0.299
	Nurse	40	18.6	4.03 ± 1.32		
	Senior nurse	77	35.8	3.57 ± 1.29		
	Nurse‐in‐charge	88	40.9	3.69 ± 1.18		
	Associate chief nurse and above	10	4.7	3.83 ± 0.89		

Marital status					0.269	0.764
	Single	67	31.2	3.77 ± 1.41		
	Married	141	65.6	3.68 ± 1.15		
	Divorced/widowed	7	3.3	3.97 ± 1.33		

Employment type					0.348	0.707
	Regular employee	37	17.2	3.79 ± 1.18		
	Contract	176	81.9	3.71 ± 1.25		
	Labor dispatch	2	0.9	3.07 ± 0.47		

ICU work experience					3.497	0.016
	< 4	58	27	3.78 ± 1.37		
	4–10	43	20	3.71 ± 1.24		
	11–14	55	25.6	4.06 ± 1.11		
	> 14	59	27.4	3.33 ± 1.13		

Monthly night shifts					0.353	0.703
	< 6	45	20.9	3.78 ± 1.15		
	6–7	109	50.7	3.76 ± 1.11		
	> 7	61	28.4	3.61 ± 1.50		

Monthly income (yuan)					0.773	0.510
	≤ 9000	36	16.7	3.65 ± 1.20		
	9001–10000	67	31.2	3.63 ± 1.15		
	10001–15000	68	31.6	3.91 ± 1.20		
	> 15000	44	20.5	3.61 ± 1.45		

### 3.2. Current Status and Correlation of Emotional Labor, Organizational Support, and Work Engagement in GICU Nurses

The scores for surface acting (2.67 ± 0.99), deep acting (3.52 ± 0.90), natural acting (3.68 ± 0.90), organizational support (3.89 ± 0.76), and work engagement (3.72 ± 1.24) were obtained. The K‐S test showed that all scales met the normality assumption.

Surface acting was negatively correlated with organizational support and work engagement, while deep acting, natural acting, and organizational support were positively correlated with work engagement (*p* < 0.05). See Table 2 for details.

**TABLE 2:** Current status and correlation of emotional labor, organizational support, and work engagement in GICU nurses.

Item	Item score ($\overset{¯}{x}$ ± SD)	1	2	3	4	5
1. Surface acting	2.67 ± 0.99	1				
2. Deep acting	3.52 ± 0.90	−0.181^∗^	1			
3. Natural acting	3.68 ± 0.90	−0.343^∗^	0.347^∗^	1		
4. Organizational support	3.89 ± 0.76	−0.439^∗^	0.153^∗^	0.303^∗^	1	
5. Work engagement	3.72 ± 1.24	−0.436^∗^	0.284^∗^	0.385^∗^	0.611^∗^	1

^∗^ *p* < 0.05.

### 3.3. Multiple Stepwise Regression Analysis of Work Engagement in GICU Nurses

Since the independent variables include categorical variables, dummy variables must be created for those with more than two categories. The reference group for each variable is set as the category with the largest sample size, specifically marital status uses “married” as the reference group, employment type uses “contract” as the reference group, professional title uses “nurse‐in‐charge” as the reference group, and education level uses “bachelor’s” as the reference group.

A multiple stepwise linear regression analysis was performed with demographic variables and organizational support, surface acting, deep acting, and natural acting as independent variables and work engagement as the dependent variable. Variables whose *F*‐test *p*‐value was < 0.05 were entered into the model, those with *p* > 0.10 were removed, and any variable whose variance inflation factor (VIF) exceeded 10 was also excluded. The results showed that organizational support, deep acting, surface acting, and natural acting were influencing factors of work engagement in GICU nurses (*p* < 0.05, VIF < 10). See Table 3 for details.

**TABLE 3:** Multiple stepwise linear regression of work engagement in GICU nurses.

Variable	*B*	*β*	*t*	*p*	VIF	*R*	*R* ^2^
Organizational support	0.788	0.481	8.319	< 0.001	1.281	0.672	0.452
Surface acting	−0.190	−0.152	−2.597	0.010	1.322		
Deep acting	0.185	0.075	2.460	0.015	1.144		
Natural acting	0.193	0.080	2.416	0.017	1.287		

### 3.4. Mediating Effect Analysis of Emotional Labor, Organizational Support, and Work Engagement in GICU Nurses

Cronbach’s alpha coefficients for the dimensions and total scores of the scales used in this study were as follows: surface acting (0.951), deep acting (0.740), natural acting (0.964), organizational support (0.982), and work engagement (0.973). All Cronbach’s alpha coefficients were above 0.7. While this indicates excellent internal consistency, a value above 0.95 may also be influenced by the high homogeneity of our sample (e.g., all participants were nurses from similar ICU settings) and warrants cautious interpretation regarding potential item redundancy. The KMO values for the scales were emotional labor (0.880), organizational support (0.947), and work engagement (0.951), all above 0.8. Bartlett’s test of sphericity was significant (*p* < 0.05), and the total variance explained was 82.736%, 86.536%, and 84.749%, respectively, indicating good validity.

**FIGURE 2:**
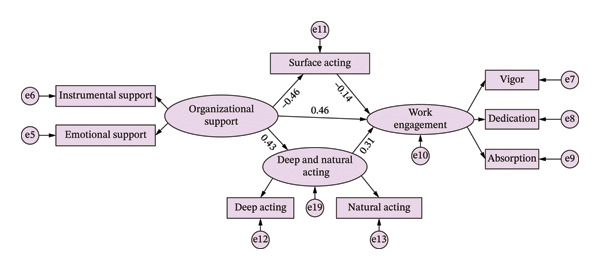
Mediating effect model of emotional labor dimensions between organizational support and work engagement; *p* < 0.05.

A structural equation model was used to form a model with organizational support as the independent variable, surface acting, deep acting, and natural acting as mediating variables, and work engagement as the dependent variable. Figure 2 presents the final structural equation model with standardized path coefficients between organizational support, the three emotional labor dimensions (surface acting, deep acting, and natural acting), and work engagement among GICU nurses. Positive coefficients indicate resource‐enhancing pathways, while negative coefficients indicate resource‐depleting pathways. All paths shown are statistically significant at *p* < 0.05. See Figure 2 and Table 4 for details.

**TABLE 4:** Mediation effects of emotional labor dimensions on the relationship between organizational support and work engagement.

	Std.	Estimate	*p*	95% CI	
				LCL	UCL
Surface acting mediation effect	0.064	0.099	0.044	0.003	0.220
Proportion of mediation effect (surface acting)	9.76% (0.064/0.656)				
Deep and natural acting mediation effect	0.136	0.211	0.003	0.055	0.556
Proportion of mediation effect (deep and natural acting)	20.73% (0.136/0.656)				
Difference between mediation effects	−0.072	/	0.232	−0.298	0.044
Total mediation effect	0.200	−0.310	0.001	0.118	0.656
Proportion of total mediation effect	30.49% (0.200/0.656)				
Direct effect	0.465	0.709	0.004	0.349	0.993
Total effect	0.656	1.018	< 0.001	0.824	1.221

The model fit results indicate that the chi‐square/degrees of freedom ratio (*χ* ^2^/df) = 2.196, root mean square error of approximation (RMSEA) = 0.075, goodness‐of‐fit index (GFI) = 0.962, adjusted goodness‐of‐fit index (AGFI) = 0.915, normed fit index (NFI) = 0.970, comparative fit index (CFI) = 0.983, incremental fit index (IFI) = 0.983, and Tucker–Lewis index (TLI) = 0.970. All these fit indices demonstrate favorable performance, indicating good model fit and reasonable conceptualization of the model structure.

The mediation effects of organizational support on work engagement were tested using the bootstrap method (sample size = 12,000, 95% bias‐corrected confidence intervals). Results showed that organizational support significantly influenced work engagement through surface acting (*β* = 0.099, 95% CI [0.004, 0.219]) and through the combined pathways of deep and natural acting (*β* = 0.211, 95% CI [0.054, 0.539]). The standardized total effect of organizational support on work engagement was 0.656. In this, surface acting accounted for 9.76% of the mediation effect, while deep and natural acting accounted for 20.73%, totaling a 30.49% mediation effect from emotional labor. Surface acting, deep acting, and natural acting jointly exerted partial mediation effects between organizational support and work engagement, confirming the validity of hypotheses H1, H2, H3, H4, H5, and H6. Therefore, surface acting and deep and natural acting should be conceptualized as parallel multiple mediators in the relationship between organizational support and work engagement.

Furthermore, the study found that the mediation mechanisms of surface acting and deep and natural acting had opposing directional effects. As organizational support increased, surface acting decreased while deep and natural acting increased, which in turn enhanced work engagement. However, the difference between the two mediation effects (Δ = −0.072, 95% CI [0.054, 0.539], *p* > 0.05) was not statistically significant^∗^, indicating that both pathways contributed equally to the overall mediation effect of organizational support on work engagement.

## 4. Discussion

### 4.1. Current Status of Emotional Labor, Organizational Support, and Work Engagement in GICU Nurses

The work engagement scores of GICU nurses were consistently lower than those reported for nurses in other studies [46–48]. This discrepancy may be attributed to the critical condition of patients in the GICU, which requires nurses to frequently manage emergency rescues and death scenarios, coupled with the heavy workload. These factors highlight the need for nursing managers to focus on and enhance the work engagement of GICU nurses [8, 49].

GICU nurses exhibit complex patterns of emotional labor management. Compared to other nurses, the current study found lower surface acting scores and higher deep acting and natural acting scores [23, 36, 50]. The relatively lower surface acting scores compared to deep and natural acting may stem from the GICU environment, where nurses have less interaction with conscious patients and their families but more interaction with colleagues. This reduces the reliance on superficial emotional responses and encourages deeper emotional management. Nurses strive to align their genuine feelings with the demands of their professional roles.

Overall, the level of organizational support for GICU nurses was above average and higher than that reported in other studies [48, 51]. This indicates that participants had a positive perception of the support provided by their organization.

### 4.2. Correlation Between Organizational Support, Emotional Labor, and Work Engagement

The results showed that organizational support was positively correlated with work engagement, surface acting was negatively correlated with organizational support and work engagement, and deep acting and natural acting were positively correlated with organizational support and work engagement, aligning with previous studies [14, 15, 22, 23]. This consistency suggests that the resource‐buffering effect of organizational support against the strain of emotional dissonance is a robust phenomenon across different professions. However, our study extends this literature by demonstrating this specific relationship in the high‐stakes, emotionally charged context of the GICU. Unlike more general settings, GICU nurses face unique emotional demands, and our finding confirms that organizational support serves as a critical resource even under these extreme conditions. In the GICU environment, nurses often face high work pressure and complex interpersonal interactions. When organizations provide sufficient support, such as emotional understanding, practical resource assistance, and managerial recognition, these factors can significantly reduce the psychological burden caused by work [52]. This is because when nurses feel supported by their organization, they no longer need to pretend to align with external expectations but can freely express emotions in a more relaxed and harmonious work environment. This change not only reduces the risk of physical fatigue and psychological exhaustion from long‐term surface acting but also fosters a more positive work attitude [53].

Perceived organizational support, reflecting employees’ sense of value to the organization, motivates nurses to adopt deeper emotional regulation strategies. When GICU nurses feel recognized and supported, they are more driven to understand and empathize with patients’ suffering, triggering positive emotional responses through cognitive reappraisal and imagination, leading to more deep and natural acting [54]. Strong organizational support also creates an environment encouraging open communication and mutual support, making it easier for nurses to seek help from colleagues, further promoting deep acting. This enhances nursing quality and strengthens nurses’ professional identity and self‐efficacy. The findings of the present study echo those of Jing et al. [55] on ethical leadership, collectively underscoring the fundamental role of organizational‐level supportive factors in shaping nurses’ positive psychological states and work behaviors. However, our research further expands this influence mechanism into the specific domain of emotional labor, elucidating how organizational support functions by empowering nurses’ emotional regulation strategies. This enriches our understanding of how a supportive environment operates.

Hospitals that prioritize fair labor distribution, career advancement, and emotional management education, and help nurses use emotional labor strategies effectively, can foster a positive organizational culture. This enables healthcare workers to better experience and express emotions in their work, respond to patients’ needs with genuine feelings, and find a connection between personal values and professional missions [56]. Within an organization characterized by care and support, nurses more readily link personal values with their professional mission, spontaneously exhibiting desirable behaviors that align with organizational expectations [57, 58]. For instance, in a GICU that emphasizes teamwork and patient primacy, nurses may naturally display patience and attentiveness by internalizing these values, without extra effort or emotional suppression. Thus, organizational support not only aids nurses in meeting work challenges but also encourages them to integrate their authentic selves into daily tasks. This reduces surface acting and increases deep and natural acting, creating a virtuous cycle that enhances job satisfaction and engagement.

### 4.3. The Parallel Multiple Mediation Effects of Emotional Labor in the Relationship Between Organizational Support and Work Engagement

The study confirms that emotional labor mediates the relationship between organizational support and work engagement in GICU nurses. In research on the mechanism of organizational support’s influence on work engagement, unlike other mediating variables such as self‐efficacy, emotional intelligence, and psychological needs that demonstrate only simple unidirectional effects, emotional labor emerges as a complex construct with double‐edged characteristics [14, 15, 48]. In assessing emotional labor, numerous studies label appropriate levels as positive while deeming excessive or insufficient levels as negative, yet fail to define precise parameters for what constitutes “appropriate.” This ambiguity stems precisely from researchers’ tendency to overlook the inherent multidimensional complexity of emotional labor, instead treating it as an undifferentiated whole [22, 59, 60]. However, studies that properly account for the intrinsic dimensions of emotional labor successfully eliminate such ambiguity [36, 61, 62]. In the bootstrap test, the proportion of the mediating effect of surface acting was 9.76%, and the combined mediating effect of deep and natural acting was 20.73%. The pathway via deep and natural acting (20.73%) was more than twice as influential as the pathway via surface acting (9.76%). This finding emphasizes that while helping nurses reduce the dissonance of surface acting is beneficial, the greater payoff for healthcare organizations lies in proactively cultivating an environment that fosters genuine emotional resonance and authenticity. Therefore, practices such as emotional intelligence training, peer support programs, and recognizing the emotional demands of nursing would likely yield a stronger return on investment than solely focusing on reducing negative emotional strategies. However, the difference between the two mediating effects was not significant, indicating that both play an equivalent mediating role in the impact of organizational support on work engagement. With adequate organizational support, surface acting decreases while deep and natural acting increases, improving the quality of emotional labor. This reduces the risk of exhaustion from maintaining false expressions and enhances nurses’ pride and sense of achievement, strengthening their professional identity and self‐efficacy [63–65]. These changes increase nurses’ willingness to invest extra time and effort in providing high‐quality care, positioning emotional labor as a key link between organizational support and work engagement.

Organizational support provides external resources through instrumental support (e.g., staffing, shift optimization, and multidisciplinary collaboration) and emotional support (e.g., psychological counseling). Nurses, via emotional labor strategies, transform these resources into adaptive emotional regulation behaviors. Surface acting drains these resources, leading to exhaustion, while deep acting adds value through cognitive reappraisal, and natural acting becomes a natural expression of internalized resources. This process of external resources being internalized and transformed into adaptive individual outcomes echoes the core finding of Qiu et al. [66], whose study demonstrated that organizational commitment fosters personal resources like psychological capital and positive coping styles, which in turn enhance professional benefit. This activates a positive “emotion‐skill” feedback loop, with work engagement serving as the outlet for resource circulation: vigor from continuous energy renewal in emotional labor, dedication from reinforced professional identity driven by organizational support, and absorption from empowerment and personal resource mechanisms [67–69]. In summary, organizational support, emotional labor, and work engagement form a resource‐strategy‐efficacy chain, with emotional labor strategies exhibiting a double‐edged sword effect—resource consumption through surface acting and positive resource conversion through deep and natural acting.

### 4.4. Strengths and Limitations

This study integrates emotional labor, organizational support, and work engagement within the JD‐R model framework, overcoming previous limitations of pairwise relationship research. By validating the mediating role of emotional labor, it reveals the progressive “resource‐strategy‐efficacy” influence pathway in GICU nurses, expanding the explanatory boundaries of this model in nursing research. Focusing specifically on GICU nurses—a high‐stress, high‐demand specialized population—the study demonstrates the double‐effect nature of emotional labor, where surface acting leads to resource depletion while deep acting and natural acting facilitate positive transformation. These findings refine the application and interpretation of emotional labor theory in ICU contexts and lay the foundation for constructing multidimensional interaction models.

This study only selected GICU nurses from some tertiary hospitals in Northeast China for convenience sampling, which may be affected by regional, hospital, and departmental factors, limiting the generalizability of the conclusions. Although the mediating role of emotional labor was verified, the impact path between organizational support and work engagement may be moderated by multilevel factors (e.g., departmental ethical atmosphere and leader–member exchange relationships). The scales used in this study were designed for all nurses and lacked specificity for GICU nurses. The use of self‐report measures for all constructs may introduce common method bias and social desirability bias; future studies should incorporate objective data, mixed methods, or multisource ratings. The cross‐sectional design prevents us from drawing definitive causal inferences. Longitudinal or experimental designs are needed in the future to establish causality. Although the multiple‐linear‐regression models controlled for the key individual‐ and hospital‐level covariates available in our dataset, variables that were not collected in the general‐information questionnaire could not be adjusted for. This leaves the study exposed to residual confounding and possible bias in the standard errors. The vast majority of existing research remains theoretical or correlational. This limitation extends beyond our study and points to a pressing need for the future direction of this research stream. Therefore, we strongly advocate for the development and implementation of evidence‐based interventions (e.g., mindfulness training, emotional skills workshops, and supervisory support programs) aimed at fostering adaptive emotional labor strategies.

## 5. Conclusion

The work engagement scores of GICU nurses were consistently lower than those reported for nurses in other studies, underscoring the urgent need for managerial attention. The dimensions of emotional labor in GICU nurses serve as multiple parallel mediators between organizational support and work engagement. Organizational support can enhance work engagement by improving emotional labor, specifically by reducing surface acting and increasing deep acting and natural acting, forming a resource‐strategy‐efficacy chain. Emotional labor strategies exhibit a double‐edged sword effect—namely, the negative consumption of surface acting and the positive transformation of deep acting and natural acting. Managers should optimize strategies by minimizing the negative impact of surface acting and enhancing the positive effects of deep acting and natural acting, thereby strengthening the positive role of organizational support on work engagement.

## Author Contributions

He Li: conceptualization, data curation, formal analysis, investigation, methodology, visualization, and writing–original draft preparation. Jing Wang: investigation, project administration, resources, and supervision. Heng Dai: resources and supervision. Hailong Hou: data curation and validation. Xianjuan Cheng: data curation and validation. Tongtong Fu: writing–review and editing. Shiqi Xiao: conceptualization, investigation, methodology, project administration, supervision, and writing–review and editing.

## Funding

This research did not receive any specific grant from funding agencies in the public, commercial, or not‐for‐profit sectors.

## Conflicts of Interest

The authors declare no conflicts of interest.

## Data Availability

The data that support the findings of this study are available from the corresponding author upon reasonable request.
